# Phase Variation During Host Colonization and Invasion by *Campylobacter jejuni* and Other *Campylobacter* Species

**DOI:** 10.3389/fmicb.2021.705139

**Published:** 2021-07-28

**Authors:** Caroline Cayrou, Natalie A. Barratt, Julian M. Ketley, Christopher D. Bayliss

**Affiliations:** Department of Genetics and Genome Biology, University of Leicester, Leicester, United Kingdom

**Keywords:** phase variation, *Campylobacter*, flagella, capsule, LPS (lipopolysaccharide), animal model, infection

## Abstract

Phase variation (PV) is a phenomenon common to a variety of bacterial species for niche adaption and survival in challenging environments. Among *Campylobacter* species, PV depends on the presence of intergenic and intragenic hypermutable G/C homopolymeric tracts. The presence of phase-variable genes is of especial interest for species that cause foodborne or zoonotic infections in humans. PV influences the formation and the structure of the lipooligosaccharide, flagella, and capsule in *Campylobacter* species. PV of components of these molecules is potentially important during invasion of host tissues, spread within hosts and transmission between hosts. Motility is a critical phenotype that is potentially modulated by PV. Variation in the status of the phase-variable genes has been observed to occur during colonization in chickens and mouse infection models. Interestingly, PV is also involved in bacterial survival of attack by bacteriophages even during chicken colonization. This review aims to explore and discuss observations of PV during model and natural infections by *Campylobacter* species and how PV may affect strategies for fighting infections by this foodborne pathogen.

## Introduction

Sporadic infections in humans or other animals have been described for 13 of the 32 *Campylobacter* species (Costa and Iraola, 2019). However, only two of these species, *Campylobacter jejuni* and *Campylobacter coli*, are frequently associated with foodborne gastrointestinal infections in humans. *C. jejuni* is considered a commensal inhabitant of gastrointestinal tracts of chickens and wild birds (Hermans et al., 2012). Consumption of contaminated poultry meat is an important source of clinical infection and therefore control of intestinal colonization of chickens forms a major strategy for the reduction of human infections (Humphrey et al., 2007). The mechanisms that enable this bacterial species to colonize its avian hosts and cause disease in humans are still not completely understood. However, the flagella, lipooligosaccharide (LOS) composition, and the capsule have been identified as being essential for colonization, invasion and survival within avian hosts (Burnham and Hendrixson, 2018). The composition of the glycans and other modifications of the flagella, LOS and capsule structures can vary markedly between *C. jejuni* strains and, interestingly, within the populations of single isolates. Most of the variability in these structures is derived from three different mechanisms: genomic recombination due to horizontal gene transfer, point mutations and phase variation (PV; Gilbert et al., 2002; Karlyshev et al.,2005a,b). Similar phenomena are known or likely to occur in the other *Campylobacter* species.

Phase variation is a phenomenon that allows bacterial populations to adapt quickly to changes in their local environment (van der Woude and Bäumler, 2004). In other bacteria, including *Neisseria meningitidis* and *Haemophilus influenzae*, PV has been identified as a mechanism allowing these bacteria to colonize their human hosts and survive immune responses (van der Woude and Bäumler, 2004; Fox et al., 2014). PV facilitates the emergence of subpopulations with diverse phenotypes even though the overall genomic content is unchanged (van der Woude and Bäumler, 2004; van der Woude, 2006; 2011). The presence of subpopulations, with a higher fitness for a range of environmental changes, increases the adaptability of these bacteria and increases survival in environmental landscapes with wide divergences in selective factors.

Studies of the contributions of PV to host colonization and invasion by *Campylobacters* have mainly focused on the phase-variable genes of two *C. jejuni* strains (NCTC11168 and 81-176). These outputs are described as an exemplar for future studies of PV in other *C. jejuni* strains and *Campylobacter* species.

## Phase Variation Mechanism in *Campylobacter* SPP.

The main features of PV are; reversibility; invariant transmission to the next generation (due to strong linkage between phenotype and genotype); stochasticity; and an occurrence of mutations at a significantly higher rate than standard mutation (van der Woude and Bäumler, 2004; van der Woude, 2011). While, there are various PV mechanisms including genomic rearrangement and differential methylation (van der Woude and Bäumler, 2004), the main mechanism observed in *C. jejuni* is slipped strand mispairing (SSM; Parkhill et al., 2000; Bayliss et al., 2012). This mechanism is based on the presence of simple sequence repeats (SSRs) in the genome. Longer length strings of repeat sequences are linked with a higher probability of an error occurring during DNA replication by mispairing of the nascent and template strands (Bayliss et al., 2012). An insertion or deletion (indel) of one repeat can be introduced in the SSR as a result of this mispairing (van der Woude and Bäumler, 2004; van der Woude, 2011). In phase-variable organisms, these SSRs are found within the open reading frame (ORF) of a given gene or within the promoter region. When the SSR is present in an ORF, indels due to changes in repeat number can introduce a frameshift, that causes a switch from a full size to a truncated form (ON to OFF) of the encoded protein or vice-versa from a truncated form to a full-size form (OFF to ON) (van der Woude and Bäumler, 2004; van der Woude, 2011). If the SSR is present in the regulatory region of the gene, switches in repeat number can affect the level of transcription of the gene. In some cases, the SSR may be present at the end of the ORF or in the termination motif of the transcript, and hence has the potential to produce a polar effect on the transcription and/or the translation of the downstream gene (Kim et al., 2012). It should be noted that indels are more likely to be maintained in *C. jejuni* SSRs after DNA replication due to the absence of a functional mismatch repair system in this species (Gaasbeek et al., 2009). The SSR indel rate in *C. jejuni* was measured at between 1 × 10^–4^ and 1.6 × 10^–3^ mutations per division (Bayliss et al., 2012). Switching from ON-to-OFF and OFF-to-ON occurs in both directions at similar rates but with a bias toward insertions in G8 tracts and deletions in tracts of G11 or longer (Bayliss et al., 2012; Aidley and Bayliss, 2014). These biases appear to have constrained *C. jejuni* tracts to mainly G9 and G10 tract lengths. These mutation-mediated switches occur continually during replication of these organisms and do not appear to be altered by environmental signals (Aidley and Bayliss, 2014).

A key feature of *C. jejuni* genomes is the presence of multiple phase-variable genes (Aidley et al., 2018). This means that a specific population of an isolate will have an identifiable SSR size pattern for each gene and for combinations of genes. These SSR sizes can be converted into expression states and these specific combinations have been termed phasotypes. The transition from one phasotype to another is called phasotype switching.

**FIGURE 1:**
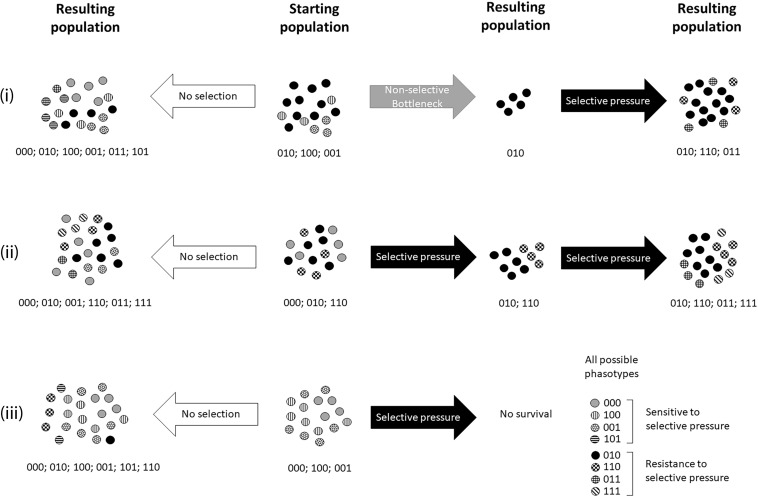
Illustration of the evolution of phasotype diversity in a bacterial population under selective and non-selective conditions. This example considers three different PV genes. These genes can all switch from an ON (1) to an OFF (0) expression state resulting in eight possible phasotypes that all have differing combinatorial expression states and where there is positive selection for the ON expression state of the second PV gene (as listed in the figure). We present three different scenarios that are all comprised of a starting population in the center that undergoes evolution with no selection **(left column)** or by selection **(right column)** with (scenario i) or without a non-selective bottleneck (scenarios ii and iii). Note that each starting population is comprised of three different phasotypes and varying proportions of each of these phasotypes. We firstly consider “No selection.” In this case, the bacterial population evolves without any selective pressure and an increase in phasotype diversity is observed in the resulting population. Differences between these three “no selection” populations reflect the stochastic nature of PV and the differing compositions of the starting populations. We next consider the three scenarios: (i) Non-selective bottleneck followed by selection. The non-selective bottleneck reduces diversity such that the resulting population only contains the major resistant phasotype and becomes the dominant phasotype following selection. (ii) Selection of partially-resistant starting population. The two resistant phasotype survive the initial cycle of selection. PV leads to appearance of the other resistant phasotypes during long-term maintenance of selection. (iii) Selection of susceptible starting population. The population does not survive due to the absence of resistant variants in the starting population. Where PV mediates survival of a selective pressure, this scenario is a rare event as PV occurs at high rates and means that most starting populations will contain phase variants of each gene.

PV generates multiple subpopulations with different phasotypes among the overall bacterial population (Figure 1). Thus, PV participates in the generation of the overall diversity of the population. This diversity is potentially important for the survival of the bacteria. For example, during a change in the environment, a subset of the population may exhibit a fitter phasotype that will have an enhanced ability to survive as compared to the major population phasotype. In this situation, the sub-population will be positively selected and will become the dominant type in the population. One result of this selective process is that the population will lose its phasotype diversity (Figure 1). Critically this diversity can be recovered over subsequent generations due to the high switching rates and if the specific selective pressure driving dominance is removed (van der Woude and Bäumler, 2004; Bayliss et al., 2012; Aidley et al., 2017b).

Interestingly, a non-selective bottleneck can also decrease phasotype diversity (Aidley et al., 2017a). During a non-selective bottleneck only a portion of the bacterial population is carried over to the new niche (Figure 1). The size of the bottleneck will determine how much phasotype diversity is lost by the population. A wide bottleneck will only have a minor or no effect on diversity, whereas a very narrow bottleneck can result in dominance by a single phasotype. Non-selective bottlenecks have been previously observed in chicken colonization experiments with *C. jejuni* and modeling has predicted that this is due to a single-cell bottleneck (Wanford et al., 2018b). This single cell will exhibit a particular phasotype and the resulting population will be dominated by this single phasotype. However, as described for positive selection, a diversification of the population will occur over the subsequent generations assuming no selective or non-selective bottlenecks re-occur. If a narrow non-selective bottleneck or environmental selection occurs the population will remain low in phasotype diversity (Aidley et al., 2017a). Non-selective bottlenecks should be taken into consideration when studying PV during *in vitro* and *in vivo* experiments to avoid misinterpretation of the functional effects of phasotype switching.

## Phase Variation in *Campylobacter* SPP.

**FIGURE 2:**
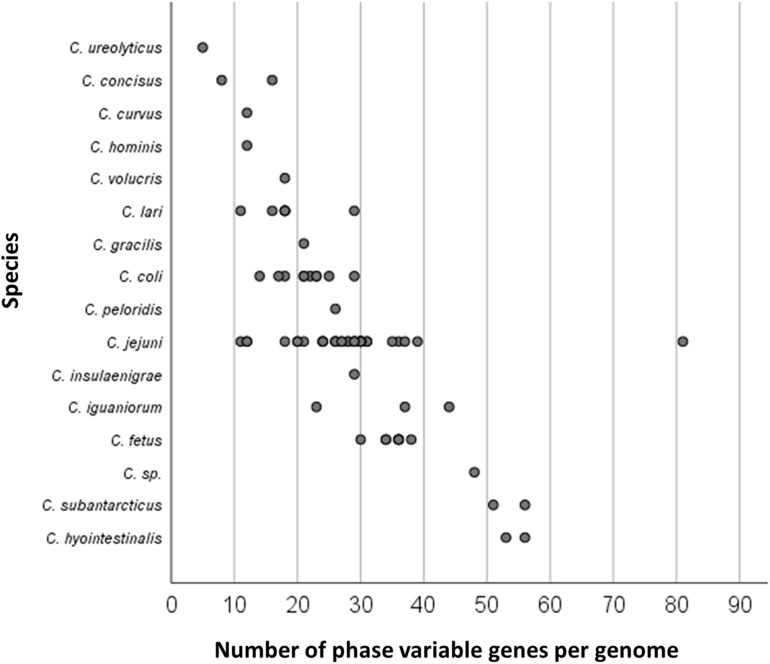
Number of PV genes per genome of 16 *Campylobacter* species. Putative PV genes were identified by the presence of a G/C homopolymeric repeat in the intragenic or intergenic region using PhasomeIt. Each circle corresponds to one isolate. The genome exhibiting more than 80 PV genes for *C. jejuni* is *C. jejuni* subsp. *doylei* strain 269.97. Adapted from Aidley et al. (2018).

The full set of PV genes (termed the phasome) of a particular isolate can be determined by whole genome sequencing. In 2000, the first complete *C. jejuni* genome was published for NCTC11168, a clinical isolate. The authors identified 32 potential PV genes within this genome sequence (Parkhill et al., 2000). A later comparative analysis of four different species (namely *C. jejuni* RM1221, *C. coli* RM2228, *Campylobacter lari* RM2100, and *Campylobacter upsaliensis* RM3195 genomes) demonstrated that homopolymeric tracts and potentially PV genes are present in a variety of *Campylobacter* species (Fouts et al., 2005). With the advent of next generation sequencing, many more genome sequences have become available and a recent snapshot found that 66,821 and 52,467 *Campylobacter* (all species) sequences were present on the NCBI and PubMLST websites (April 2021), respectively (Jolley et al., 2018; Sayers et al., 2021). Of these, 64,121 (NCBI) and 51,279 (PubMLST) belong to *C. jejuni* and *C. coli* species. The origins of these samples are diverse but are dominated by isolates from human and chicken samples. Until recently, it was difficult to determine and compare the phasomes of large genome datasets. This problem was overcome by development of a new tool, PhasomeIt, for identifying genomic PV-associated SSRs sequences. Successful application of this tool to the genomes of the *Campylobacter* (Figure 2; Aidley et al., 2018) and *Neisseria* genera (Wanford et al., 2018a) demonstrated that the number of phase-variable genes varies both among and within species. For instance, the number of potential PV genes ranges from 18 to 39 among *C. jejuni* strains. In comparison, one *Campylobacter* species, *Campylobacter ureolyticus*, had <5 phase-variable genes while two, *Campylobacter hyointestinalis* and *Campylobacter subantarticus*, had > 50. For the majority of *Campylobacter* spp., PV is based on the presence of G/C homopolymeric tracts present in ORFs, but with a consistent minority in the intergenic regions (Aidley et al., 2018). An unexplored phenomenon is the presence of homopolymeric A/T or dinucleotide TA repeat tracts in some putative *Campylobacter* PV genes (Miller et al., 2005; Aidley et al., 2018). The wide distribution of SSRs in this genus suggests that evolution of PV in *Campylobact*er species is partly driven by molecular aspects of DNA metabolism or genome composition.

A key feature of PhasomeIt was the classification of PV genes into homology groups. This classification is based on a threshold level of protein sequence homology between PV genes and other PV or non-PV genes (see Aidley et al., 2018 for more details). The groups are produced in a network fashion such that two non-homologous PV genes can be in the same group due to high homology with a third gene. The vast majority (∼95%) of these groups were found in only a few isolates indicative of weak selection and a high turnover. The temporary nature of PV was further reflected in the frequent presence of non-phase-variable genes within a homology group. A prime example was the restriction-modification (RM) genes of the *cj0031* group that were only phase-variable in a small number of *C. jejuni*, *C. coli*, and *C. lari* strains despite being almost invariably present (Aidley et al., 2018). Aidley et al. (2018) also explored whether there were species-specific functional conservation of phase-variable genes. These genes were termed the core phasome and were defined as genes that were present in >60% of isolates. The four major species (*C. jejuni*, *C. coli*, *Campylobacter fetus*, and *C. lari*) were observed to have species specific core phasomes of 2–27 homology groups but with *C. jejuni* and *C. coli* sharing five homology groups in their core phasomes (namely, *cj0045c*, *cj0170*, *cj0617*, *maf1*, and *cj1295*; Aidley et al., 2018). Intriguingly 17 homology groups were found in multiple species with the *cj1295* homology group being present in 10.

Another key finding by Aidley et al. (2018) was that most of the major homology groups consisted of transferases or enzymes with roles in modifications of LOS, capsular polysaccharides, or the flagellum. This over-representation of these gene classes highlights the potentially important role played by PV in modulating the functions of these major surface structures and the contributions of these structures to bacterial interactions with host organisms.

Among the isolates, analyzed by Aidley et al. (2018), are present four of the most commonly used *C. jejuni* laboratory isolates. Definition of the phasomes of these isolates is important due to frequent use of these strains for exploring mechanisms of intestinal colonization, invasion of host cells and other aspects of *Campylobacter* biology. The four genomes of the M1, NCTC11828, 81–176, and NCTC11168 isolates contain 12, 18, 20, and 31 phase-variable genes, respectively. In addition, the PV genes identified in these isolates cover 13 of the 20 major homology groups (Aidley et al., 2018). Most of these phase-variable genes are present within the genomic clusters of flagellum, capsule, and LOS biosynthetic genes (Supplementary Table 1). Thus the phasomes of these laboratory isolates are representative of the diversity of PV gene numbers for *C. jejuni* genomes and are useful tools for studying the biological functions of PV genes.

## Functions of the *C. jejuni* Flagella, Capsule and Los-Associated Phase-Variable Genes

The *Campylobacter* genomic region encoding genes required for flagella formation and glycosylation contains the largest number of phase-variable genes (Aidley et al., 2018; Supplementary Table 1). *Campylobacter* motility plays an important role in intestinal colonization and host cell invasion (Guerry, 2007). *C. jejuni* motility relies on the presence of one or two polar flagella. The flagellum is embedded in the membrane via a basal body connected by a hook to a long polymeric filament mainly composed of the flagellin subunit FlaA (Cohen et al., 2020). The expression of the flagellar genes is controlled by δ^54^ and δ^28^ factors, the FlgSR two-component system, FlhF (a putative GTPase) and the flagellar export apparatus (Hendrixson and DiRita, 2003; Balaban et al., 2009; Joslin and Hendrixson, 2009). In addition, the *C. jejuni* flagellum is extensively glycosylated (Nuijten et al., 1995; Thibault et al., 2001). PV alters the expression and modifications of the flagellum at various levels. The FlgSR system expression, and hence motility, can be controlled by a high frequency of non-reversible mutations in poly A/T tracts in both *flgR* and *flgS* (Hendrixson, 2006, 2008). The *flgR* and *flgS* repeat tracts consist of less than 7 repeats and hence the mutations are not reversible and are probably observed due to very strong selective pressures. The Cj1313 homology group is part of the flagellar glycosylation pathway but also appears to be involved in bacterial motility as deletion of the *pseH* gene reduces motility in the 81–176 isolate (McNally et al., 2006). Three other homology groups, *maf1*, *cj0170*, and *cj1295*, also encode enzymes involved in flagellar glycosylation. Changes in expression of these genes alter the glycosylation pattern of the flagellum as observed during gene deletion studies in *C. jejuni* and *C. coli* (McNally et al., 2007a; van Alphen et al., 2008; Hitchen et al., 2010). In addition, the deletion of *maf4* (part of the *maf1* homology group) in the laboratory isolate 108, negatively affected auto-agglutination (van Alphen et al., 2008).

The locus encoding capsule biosynthesis genes has the second highest number of phase-variable genes (Aidley et al., 2018; Supplementary Table 1). The capsule is involved in host cell invasion and resistance to complement-mediated killing (Bacon et al., 2001). The capsule locus is organized into three regions based on function and the level of variability. Regions one and three contain genes involved in capsule assembly/transport and are highly conserved, while region 2 is highly variable, with widely differing numbers of genes, and is responsible for the synthesis of the capsule polysaccharide (Guerry et al., 2012). In addition, region 2 often contains phase-variable genes that can add non-essential modifications to the capsule. The function of three of the capsular phase-variable genes has been identified with *cj1421c* and *cj1422c* being 6-*O*-methyl phosphoramidate (MeOPN) transferases and *cj1426c* having 6-*O*-methyl transferase activity (McNally et al., 2007b; Sternberg et al., 2013). The other capsular phase-variable genes have homology to glycosyl transferases, but their exact role in capsular glycosylation still needs to be determined.

Detailed genetic and functional analyses have shown that 18 of the 19 LOS classes present in *C. jejuni* strains contain 1 or 2 phase-variable genes and that these genes can alter a variety of LOS structures (Parker et al., 2008; Houliston et al., 2011). Aidley et al. (2018) identified three major homology groups associated with LOS modification (i.e., *CJJ81-176-1160*, *wlaN* and *cj1144c*; Supplementary Table 1). The ABC LOS classes of *C. jejuni* have been associated with a rare neurological disorder, Guillain-Barre syndrome (GBS), and other polyneuropathies (Allos, 2001; Hameed et al., 2020). GBS is due to molecular similarity between ganglioside-like epitopes in the outer core of the LOS and human gangliosides. *C. jejuni* LOS can mimic GM1, GM2, GM3, GD1a, GI1a, and Gq1b structures and four phase-variable genes, *cgtA, cgtB, wlaN* (*cj1139*), and *cj1145*, are associated with this mimicry (Moran and Prendergast, 2001; Semchenko et al., 2010; Guirado et al., 2020). Linton et al. (2000) showed for the NCTC11168 strain that the *wlaN* genes encodes a β-1,3-galactosyltransferase; the *wlaN* ON state is associated with production of a GM1-like LOS whereas the OFF state generates a GM2-like structure. The *cgtB* gene is also a β-1,3-galactosyltransferase and catalyzes a similar activity. Critically the *wlaN* and *cgtB* genes are differentially distributed with *cgtB* being associated with wild bird isolates and *wlaN* with human and chicken broiler *C. jejuni* isolates (Guirado et al., 2020). Additionally, Guerry et al. (2002) showed that *cgtA*, which encodes a N-acetylgalactosaminyl (GalNAc) transferase, is responsible for a switch between GM3-like and GM2-like structures in *C. jejuni* strain 81–176 (Guerry et al., 2002). The last phase-variable gene, *cj1145*, is reported to encode a putative α-1,4 galactosyltransferase and it has been observed that an OFF state resulted in the absence of terminal α-linked galactose units in the LOS structure (Semchenko et al., 2010). However, the exact function of this gene in the context of LOS structure remains to be confirmed.

## The Function of the Other Phase-Variable Genes

As described earlier a number of phase-variable genes in *C. jejuni* are not associated with flagellar, capsule or LOS biosynthesis (Aidley et al., 2018; Supplementary Table 1). The functions of five of these genes have been explored. The *cj0031* gene is predicted to encode a Type IIG RM system and has been shown to regulate the expression of 219 genes (>1.5-fold) in *C. jejuni* strain NCTC11168 (Anjum et al., 2016). This gene also mediates resistance to phage infection (Anjum et al., 2016). A novel PV phenomenon may be occurring for the SSR in *cj0045* as this SSR is suspected to modulate the level of expression of the downstream gene, *cj0044* (Kim et al., 2012). Both genes are potential virulence factors as PV of *cj0045* has been associated with changes in invasion and colonization of mice while *cj0044* has been linked to alterations in motility due to its proximity to three genes involved in flagella formation, *cj0041, cj0042*, and *cj0043* (Kim et al., 2012). Two other virulence factors are also phase-variable. Inactivation of the phase-variable chaperone *cj0175* (*clpX*) affects *C. jejuni* survival at 42°C, auto-agglutination and invasion of cell lines (Cohn et al., 2007). Similarly, a phase-variable autotransporter, *cj0628* (*capA*), was shown to contribute to adhesion and invasion and to be necessary for chicken colonization (Ashgar et al., 2007). Several of the other phase-variable genes, including *cj0046*, *cj0565*, and *cj0676*, are annotated as pseudogenes in some strains. For these genes there is uncertainty as to whether PV can enable production of full-length proteins and hence careful analysis in a range of strains is required to understand their functions and biological roles.

## Observations of PV in Infection Models

There are two important points to take into consideration during the analysis of PV especially during *in vivo* studies. The first one is that the starting population phasotype is important for the observation of a role for PV in host adaptation. Indeed, if the inoculum population is not diverse enough and a gene is already in a favorable phasotype, no change will be observed after colonization and hence selection for specific states of important phase-variable genes may be missed. The second point is the effect of non-selective bottlenecks. A particular phasotype may appear to have been selected by the host passage but in fact a non-selective bottleneck occurred and artificially increased the frequency of the phasotype. This is particularly likely if the phasotype is already relatively common in the inoculum population.

No standard method exists to explore the biological roles of PV. One approach is to explore the alterations in *C. jejuni* phase-variable genes during colonization and invasion using animal models and cell lines. The majority of these studies have used *in vitro* cell invasion or chicken and mouse models of intestinal colonization. Chickens are the natural reservoir of *C. jejuni* and can be used to explore the contributions of PV to colonization of the avian gut (Semchenko et al., 2010; Bayliss et al., 2012; Kim et al., 2012; Wanford et al., 2018b). Another animal model is C57BL/6J IL -/- mice, this is an imperfect model of human enteritis (Jerome et al., 2011; Kim et al., 2012; Artymovich et al., 2013; Revez et al., 2013; Thomas et al., 2014). In addition to laboratory animal models, PV has been directly explored by exploiting cases of accidental laboratory infections of humans (Revez et al., 2013; Thomas et al., 2014) and a human volunteer study (Crofts et al., 2018). The human studies explored if colonization induced a change in the phasotype compared to the original laboratory strains or the inoculum. Crofts et al. (2018) complemented the human volunteer model with a primate model.

The possibility of a host-specific phasotype change has been explored. Thomas et al. (2014) used human-adapted isolates to infect mice and to determine if the phasotype changes during mouse passage. Kim et al. (2012) considered a similar possibility with chicken-adapted isolates and infection of chickens and mice, but with a focus on the influence of the phasotype on infection of a new host and changes on re-infection of the host. The use of NCTC11168 as the infecting strain in both studies facilitates comparisons as the results are not biased by potential differences in the genomic background of the test strain.

In Table 1, we consider the data from six *in vivo* studies of which three used single strains (i.e., NCTC11168 or 81–176) and the other compared NCTC11168 with six clinical isolates (Semchenko et al., 2010; Jerome et al., 2011; Bayliss et al., 2012; Kim et al., 2012; Artymovich et al., 2013; Revez et al., 2013; Thomas et al., 2014). We see that 24 PV genes exhibited a repeat size change during chicken, murine or human host passage (Table 1). Interestingly, the majority of the genes in which variation was observed were associated with the flagellar, capsule and LOS biosynthesis loci. Unfortunately, it is difficult to definitively conclude if some phase-variable gene expression states are specific to a particular host as divergent outcomes were observed and because the starting populations had different phasotypes. However, these studies seem to indicate that particular phasotypes are host specific. Thomas et al. (2014) noticed that phasotypes obtained after a human passage are not necessarily maintained during mice infection (Thomas et al., 2014). A key example was the pseudogene, *cj0046*, which exhibited a change in the frequencies of specific repeat tract lengths after colonization of mice whereas the frequencies were not changed following human passage. In addition, Kim et al. (2012) observed that some phasotypes were not essential for chicken colonization but did exhibit essentiality for colonization of mice. Specifically, these authors observed that the bacteria weren’t able to colonize mice if the chicken-adapted populations contained only the ON phasotypes of *cj0045*, *cj0685* (*cipA*), *cj1139*, *cj1421*, and *cj1426*.

**TABLE 1:** Phase variation genes potentially involved in host colonization and infection.

Genes	Repeat Size Change after Host Passage^ab^			Bacterial Structure or Function
	**Chicken^c^**	**Mice^d^**	**Human^e^**	
*cj0170*		+*	+	Flagella
*cj0685*	+*	+	+*	
*cj1295*		+*	+	
*cj1296*		+*	+	
*cj1305*		+	+	
*cj1306*		+*	+*	
*cj1310*			+	
*cj1321*			+	
*cj1325*		+*	+	
*cj1342*		+*	+	
*cj1420*			+	Capsule
*cj1421*		+		
*cj1422*		+		
*cj1426*		+	+	
*cj1429*		+*		
*cj1437*			+	
*cj1139*	+*	+	+*	LOS
*cj1145*	+	+*	+*	
*cj0031*	+*	+*	+	Type II restriction modification
*cj0045*		+*	+*	Iron binding
*cj0275*			+	Protease-clpX
*cj0628*		+	+	capA
*cj0676*		+*	+	Potassium transport-kdpA
*cj0565*		+*		Pseudogene (hypothetical protein)
*cj0046*		+*	+	Pseudogene (putative sodium:sulfate transmembrane transport protein)

*^*a*^+a change of repeat tract length has been observed by the authors after host passage*

^b^*A significant difference of the phasotype frequency has been observed.

*^*c*^Passage of NCT11168, 81–176 and six clinical isolates into chicken host (Semchenko et al., 2010; Bayliss et al., 2012).*

*^*d*^NCTC11168 isolate passage through C57BL/6J IL -/- mouse model (Jerome et al., 2011; Kim et al., 2012; Artymovich et al., 2013; Thomas et al., 2014).*

*^*e*^Accidental human infection with NCTC11168 isolate (Revez et al., 2013; Thomas et al., 2014).*

Among the 24 loci with a potential role in colonization, statistical analysis highlighted 15 phase-variable genes with a significant change in expression state after host passage (Jerome et al., 2011; Kim et al., 2012; Artymovich et al., 2013; Revez et al., 2013). PV of *cj0170* and *cj0045* to ON and OFF states, respectively, were strongly associated with murine infection (Kim et al., 2012; Artymovich et al., 2013). Switches in 11 additional phase-variable loci were significantly associated with mouse passage, with the loci *cj0031*, *cj0046*, *cj0676*, *cj1295*, *cj1296*, *cj1325*, and *cj1429* exhibiting enrichment of the ON phasotype after mouse passage and the *cj0045*, *cj0170*, *cj1145*, *cj1306*, and *cj1342* genes being enriched for an OFF phasotype (Jerome et al., 2011). In humans, only five loci exhibited a significant change of phasotype, with *cj1139*, *cj1144*, *cj1306*, and *cj0456* exhibiting an increase of the ON phasotype and *cj0045* showing an increase of the OFF phasotype (Revez et al., 2013).

## The Role of Phase-Variable Genes During Cell Invasion and Colonization of the Host

As shown in Table 1, the majority of the phase-variable genes associated with host colonization and invasion belong to the flagellar, capsule and LOS biosynthesis loci. One of the major genes associated with mouse colonization is *cj0170*. This gene is involved in motility and potentially in the glycosylation of the flagellum (Artymovich et al., 2013). Interestingly, a *cj0170* ON phasotype is associated with initial mouse colonization but after mouse passage the OFF phasotype is selected. This difference could be explained by the fact that after colonization a different PV gene is selected and *cj0170* is no longer required.

The other genes belonging to the flagellar, capsule and LOS biosynthesis loci and associated with chicken, mouse and human colonization are linked to glycosylation of these structures. The combination of phasotypes of these genes will generate a particular pattern of glycosylation on the bacterial surface. Three examples of phase-variable *C. jejuni* glycans with functional effects are flagellar legionaminic acid glycans, capsular MeOPN glycosylation and LOS sialylation; addition of these glycans affects host colonization, serum resistance and survival in the host (Guerry et al., 2002; Howard et al., 2009; Pequegnat et al., 2017). This suggests that one role of these phase-variable genes during colonization of the host is to confer the ability to change and adapt surface glycosylation patterns resulting in enhanced avoidance of innate immune effectors or adaptive immune response.

The role of non-flagella, capsule and LOS phase-variable genes during colonization is less clear. For instance, the phase-variable gene, *cj0045* encodes a putative iron binding protein, but the homopolymeric tract is situated at the 3′ end of the gene and therefore only slightly alters the length of the protein. As discussed above, it is possible that PV does not affect the function of *cj0045* but alters expression of the downstream gene, *cj0044* (Kim et al., 2012). Analysis of the gene sequences indicates that the non-truncated version of the *cj0045* ORF overlaps with the *cj0044* coding sequence. This arrangement may either allow for more efficient coupling of the translation of the two genes from a polycistronic mRNA or may decrease translation due to the overlap of the stop codon of the *cj0045* non-truncated version with the *cj0044* start codon (Kim et al., 2012). The function of *cj0044* is unknown, but the adjacent gene, *cj0043 (flgE)*, is involved with flagella formation as it encodes the flagellar hook protein and so it is possible that *cj0044* is also involved in motility (Kim et al., 2012).

Two pseudogenes in NCTC11168, *cj0046* and *cj0676*, are potentially involved in colonization and invasion. The functions of both genes are unknown but both were identified as transporters by homology analysis. The *cj0046* gene could be a potential sodium sulfate transporter whereas *cj0676* may be a potential potassium transporter. Both genes may be linked to metabolism and have a role in surviving changing environmental conditions for other *C. jejuni* strains where the genes are intact.

The last phase-variable gene associated with host colonization is *cj0031*. As previously described, *cj0031* encodes a Type IIG RM system. It has been observed that strains not expressing *cj0031* due to PV or deletion have decreased adhesion and invasion levels in a Caco-2 cell line (Anjum et al., 2016). In addition, the proportion of the OFF variants was significantly reduced in the bacterial population after passage of the NCTC11168 strain through the chicken host (Bayliss et al., 2012). As it is unlikely that this system mediates direct interactions with the cells, its role during host colonization is probably linked to a phase-variable regulatory function mediated by changes in DNA methylation of regulatory sequences of specific genes, termed a phasevarion, as observed in this and other species (Srikhanta et al., 2005; Atack et al., 2015; Anjum et al., 2016).

Importantly, the contributions of a number of potentially phase-variable genes to colonization and invasion have been characterized by gene deletion studies. Deletion of *cj0031*, *cj0628* (*capA*), and *cj0685* (*cipA*) induced significant decreases in invasion of cell lines by the mutants compared to the wild-type (Ashgar et al., 2007; Javed et al., 2010; Anjum et al., 2016). In addition, deletion of *capA* has been shown to decrease colonization of the chicken gut (Ashgar et al., 2007). Conversely, deletion of *cgtA* was shown to increase invasion (Guerry et al., 2002). These studies do not illustrate a direct role for PV of the gene in invasion and colonization, but highlight the potential role of switching OFF of the specific phase-variable genes. These types of studies need to be complemented by experiments where switching is observed or by use of strains where the repeat tract is locked ON so no PV can occur and the opposing phenotype to the deletion mutant is observed.

## Impact of Phase Variation on *Campylobacter* Mitigation Strategies

*Campylobacter* mitigation strategies are still in the developmental stage and are highly varied with most of these proposed interventions spanning pre- and post-processing of poultry (Soro et al., 2020). The potential impact of PV on these strategies has not been explored in any detail, but PV is most likely to interfere with biological interventions rather than physical or metabolic approaches. A key idea underpinning this statement is that PV may interfere with binding of killing molecules (e.g., bacteriocins, phage receptors or antigen-specific antibodies) by altering the specific attachment site or the overall surface-charge of the bacterial surface. One example of where PV was examined was for a *C. jejuni* vaccine targeting a conserved N-linked protein glycan (Nothaft et al., 2017). No consistent PV differences were detected between vaccinated and non-vaccinated birds following challenge indicating that PV was not mediating resistance to the immunity elicited by the vaccine. The complex nature of these experiments was highlighted by observations of high levels of bird-to-bird variation in PV states probably arising as a result of an inoculation bottleneck (Wanford et al., 2018b). This example serves as a model for how interference of PV with a mitigation strategy can be tested during development of these strategies.

## Phase Variation and Phage

Resistance to bacteriophage infection is one of the major potential roles of phase-variable genes in *campylobacters* that has been recently explored. This aspect is especially important as phage therapy is being developed as a mechanism to control *C. jejuni* colonization in chicken broilers (Janež and Loc-Carrillo, 2013). In order to infect their bacterial target, bacteriophages need to recognize a specific structure present on the surface of the bacteria. After binding to the surface, the phages can inject their genetic material and start the production of new viral particles. Two strategies adopted by bacteria to avoid phage infection are relevant to PV, the first is to modify their surface structure to prevent phage recognition and binding. The second strategy is to eliminate the phage genetic material once it has entered the cell. *C. jejuni* utilizes both strategies and an example is PV of genes present in the capsule locus (Holst Sørensen et al., 2012; Aidley et al., 2017b). Three genes, *cj1421*, *cj1422*, and *cj1426* have been identified as major phase-variable genes for conferring resistance to infection by phage F336 with either *cj1421* switching to an OFF state or *cj1422* switching into an ON state to survive infection. If the phage is able to inject its DNA inside the bacterial cell, an RM system can confer resistance to the phage by eliminating the foreign DNA. Among *C. jejuni* phase-variable genes, as stated previously, *cj0031* encodes for an RM system and when expressed confers resistance to infection by some phages (Anjum et al., 2016).

Phages do not only recognize capsular glycans, but also bind to flagellar glycans (Coward et al., 2006). While several phase-variable genes are involved in flagellum biosynthesis and flagellar glycosylation, only specific capsule glycosylation phase-variable genes have been identified as being involved with phage resistance and so further work is required to determine which, if any, flagellum-associated phase-variable genes are also involved in phage resistance.

The existence of PV-based mechanisms involved in the generation of phage resistance in *C. jejuni* might prevent or reduce the efficacy of any phage-based treatment and should be taken in to consideration during the development of any phage treatment. Two different approaches could be used to overcome possible phage resistance due to PV. The first is to avoid phages that target structures modulated by PV genes. This can be very difficult as the major glycosylated structures of *C*. *jejuni* (flagellum, capsule, and LOS) are all partially phase-variable. Alternatively, phage combinations, chosen to cover different PV-modified targets, could be combined to reduce resistance to phage action by *C. jejuni*. Or, the phages selected could target the ON and OFF states of the same gene which would avoid escape through PV. Finally, an important aspect of developing any phage-based therapy will be a clear understanding of the roles of PV genes in phage resistance in the context of their role in colonization and host-cell interactions.

## Conclusion

The various host colonization studies exploring PV have shown that PV is at least partially involved in the colonization process, with *cj0170* and *cj0045* being strongly associated with host colonization. Other phase-variable genes, especially those associated with the flagella, capsule and LOS loci, are implicated in colonization of the host. We note however that care should be taken when extrapolating from animal infection model results to human infections due to the significantly differing molecular and immunological differences in these niches. Indeed, phase-variable gene phasotypes favorable to colonization are potentially host-specific. The majority of studies have only explored PV and colonization with the well-characterized *C. jejuni* isolate NCTC11168, however, the very diverse genomic backgrounds among *C. jejuni* strains may influence the way phase-variable genes are involved in colonization. In addition, genes are not necessarily phase-variable in all the isolates even within in a species (Aidley et al., 2018). These phasome differences raise concerns about extrapolation from the current literature to all *C. jejuni* isolates and to other *Campylobacter* species. Thus some of the genes identified as important may have no effect in other strains while other PV genes may have major effects on colonization and invasion of hosts or during disease states in zoonotic infections. Despite increasing insights into the role of PV during colonization, further analysis is required to confirm and understand the precise role played by individual PV genes and combinatorial PV states. This information will be important for determining the potential for improving the effectiveness of therapeutic approaches.

## Author Contributions

CC wrote the review. NB reviewed and revised the text. JK and CB conceived the idea and reviewed the text. All authors contributed to the article and approved the submitted version.

## Conflict of Interest

The authors have an on-going collaboration with MERCK who are part of the collaborative project with which this article is linked.

## Publisher’s Note

All claims expressed in this article are solely those of the authors and do not necessarily represent those of their affiliated organizations, or those of the publisher, the editors and the reviewers. Any product that may be evaluated in this article, or claim that may be made by its manufacturer, is not guaranteed or endorsed by the publisher.
