# Supplementary material for: Phase Variation During Host Colonization and Invasion by Campylobacter jejuni and Other Campylobacter Species
Source: Front Microbiol. 2021 Jul 28;12:705139. doi: 10.3389/fmicb.2021.705139 (PMC8355987; doi:10.3389/fmicb.2021.705139)
Supplement: Supplementary file 1 [file Table_1.docx]

Supplementary Table 1. Phase variable genes identified for the four major C. jejuni laboratory isolates.

| **Main structures affected** | **Homology group name** | **Likely Function** | **Isolates and genes identified (clonal complex)^a^** | | | |
| --- | --- | --- | --- | --- | --- | --- |
|  |  |  | **NCTC 11168**  **(ST-21)** | **81-176 (ST-42)** | **81116; NCTC 11828 (ST-283)** | **M1 (ST-45)** |
| flagella | maf7* | Carbonic anhydrase | **Cj0617**  **Cj1305c**  **Cj1306c**  **Cj1310c**  **Cj1342c** | **CJJ81176_0646**  **CJJ81176_1327**  **CJJ81176_1341**  **CJJ81176_1321** | **C8J_1243**  **C8J_1258** | **CJM1_1282**  **CJM1_1299** |
|  | cipA* | Invasion protein CipA | **Cj0685c** | **CJJ81176_0708** | **C8J_0652** | **CJM1_0667** |
|  | Cj1295* | Amino peptidase associated with modification of pseudominic acid | **Cj1295** | **CJJ81176_1312**  **CJJ81176_1313** | **C8J_1238** | **CJM1_1277** |
|  | pseH | UDP-4-amino-4,6-dideoxy-beta-L-AltNAc o-acetyltransferase | *Cj1313* | **CJJ81176_1330** | **C8J_1246** | **CJM1_1285** |
|  | Cj1296* | Aminoglycoside N3'-acetyltransferase | **Cj1296**  *Cj1298* | *CJJ81176_1315* | *C8J_1239* | *CJM1_1278* |
|  | maf1* | Motility accessory factor | **Cj1318**  **Cj1335**  *Cj1333* | *CJJ81176_1336* | *C8J_1253* | *CJM1_1292* |
|  | Cj0170* | SAM-dependent methyltransferase | **Cj0170**  **Cj1325** | **CJJ81176_0206** |  |  |
|  | epsM* | Putative transferase | **Cj1321** |  |  |  |
| capsule | ubiE_3* | SAM-dependent methyltransferase | **Cj1420c** | **CJJ81176_1419** | **C8J_0641** | **CJM1_0659** |
|  | kpsF | Arabinose-5-phosphate isomerase | ***Cj1443c*** | *CJJ81176_1437* | **C8J_1350** | *CJM1_1390* |
|  | Cj1421c* | MeOPN transferase | **Cj1421c**  **Cj1422c** | **CJJ81176_1421**  **CJJ81176_1435** |  |  |
|  | Cj1429c | Hypothetical protein Cj1429c | **Cj1429c** | **CJJ81176_1429** |  |  |
|  | hyaD_3 | Galactosyltransferase | *Cj1440c* | **CJJ81176**_**1432**  *CJJ81176_1433* |  |  |
|  | Cj1426c | 6-O-Me transferase | **Cj1426c** |  |  |  |
|  | Cj1437c | Aminotransferase | **Cj1437c** |  |  |  |
| LOS | CJJ81176_1160 | Beta-1,4-N-acetylgalactosaminyltransferase | *Cj1143* | **CJJ81176**_**1160**  *CJJ81176_1154* | *C8J_1086* | *CJM1_1124* |
|  | wlaN | Beta-1,3 galactosyltransferase | **Cj1139c** |  |  |  |
|  | Cj1144c | Putative alpha-1,4 galactosyltransferase | **Cj1144c** |  |  |  |
| Others | hxuB_1* | Heme/hemopexin transporter protein HuxB precursor | **Cj0742** | **CJJ81176**_**0765**  *CJJ81176_0764* | **C8J_0706** | **CJM1_0723** |
|  | Cj0045c* | Hemerythrin-like iron-binding protein | **Cj0045c** | *CJJ81176_0083* | **C8J_0048** | **CJM1_0054** |
|  | CJJ81176_0758 | Putative periplasmic protein | *Cj0735* | **CJJ81176**_**0758** | **C8J_0703** | **CJM1**_**0719** |
|  | Cj0067 | Chlorohydrolase | **Cj0067** | **CJJ81176_0105** | *C8J_0060* | *CJM1_0069* |
|  | paeR7IM | Type II restriction endonuclease | **Cj0031** | *CJJ81176_0068* | *C8J_0034* | *CJM1_0039* |
|  | clpX | ATP-dependent Clp protease ATP-binding subunit ClpX | **Cj0275** | *CJJ81176_0302* | *C8J_0252* | *CJM1_0259* |
|  | kdpA | Pseudogene (potassium-transporting ATPase A chain) | **Cj0676** | *CJJ81176_0699* | *C8J_0628* | *CJM1_0645* |
|  | CJJ81176_0082 | Conserved domain protein | *Cj0044c* | **CJJ81176**_**0082** | *C8J_0047* | *CJM1_0053* |
|  | CJM1_0674 | Probable membrane protein | *Cj0692c* | *CJJ81176_0715* | *C8J_0659* | **CJM1_0674** |
|  | CJJ81176_0590 | FIG00470712: hypothetical protein |  | **CJJ81176**_**0590**  *CJJ81176*_0591 | **C8J**_**0526** | **CJM1**_**0540** |
|  | lgrA* | Formyl transferase domain protein |  | **CJJ81176_1325** | *C8J_1241* | *CJM1_1280* |
|  | BN867_11290 | dTDP-6-deoxy-3,4-keto-hexulose isomerase |  |  | **C8J_1078** | **CJM1_1116** |
|  | vacA | Autotransporter |  |  | **C8J_1278** | *CJM1_1321* |
|  | C8J_0464 | Hypothetical protein |  |  | **C8J_0464**  **C8J_0909** | *CJM1_0478*  *CJM1_0945* |
|  | Cj0046 | Pseudogene (putative sodium:sulfate transmembrane transport protein) | **Cj0046** |  |  |  |
|  | Cj0565 | Pseudogene (conserved hypothetical protein) | **Cj0565** |  |  |  |
|  | Cj0628* | Putative lipoprotein-capA | **Cj0628**  *Cj1677* |  |  |  |
|  | CJJ81176_0086 | Anion transporter |  | **CJJ81176_0086** |  |  |
|  | BN867_11310 | dTDP-6-deoxy-3,4-keto-hexulose isomerase |  |  | **C8J_1080** |  |
|  | C8J_1334 | Hypothetical protein |  |  | **C8J_1334** |  |
|  | C8J_1341 | Hypothetical protein |  |  | **C8J_1341** |  |
|  | Total number of phase variable genes | | 31 | 20 | 18 | 12 |
| Data where extracted from Aidley et al. (2018) and include only data for homopolymeric repeat tract based phase variable genes.  ^a^ The gene names in bold indicate phase variable genes and gene names in italic indicate non-phase variable homologous genes.  *Homology group belonging to the 20 major homology groups (>19 PV genes) | | | | | | |
